# Transition Metal Sensing with Nitrogenated Holey Graphene: A First-Principles Investigation

**DOI:** 10.3390/molecules28104060

**Published:** 2023-05-12

**Authors:** Uroosa Sohail, Faizan Ullah, Nur Hazimah Binti Zainal Arfan, Malai Haniti Sheikh Abdul Hamid, Tariq Mahmood, Nadeem S. Sheikh, Khurshid Ayub

**Affiliations:** 1 Department of Chemistry, COMSATS University Islamabad, Abbottabad Campus, Abbottabad 22060, Pakistan; uroosasohail6666@gmail.com (U.S.); faizan@faizanullah.com (F.U.); mahmood@cuiatd.edu.pk (T.M.); 2 Chemical Sciences, Faculty of Science, Universiti Brunei Darussalam, Jalan Tungku Link, Gadong BE1410, Brunei; 22m1432@ubd.edu.bn (N.H.B.Z.A.); haniti.hamid@ubd.edu.bn (M.H.S.A.H.); 3 Department of Chemistry, College of Science, University of Bahrain, Isa Town 32038, Bahrain

**Keywords:** nitrogenated holey graphene, sensors, density functional theory, QTAIM, electronic properties

## Abstract

The toxicity of transition metals, including copper(II), manganese(II), iron(II), zinc(II), hexavalent chromium, and cobalt(II), at elevated concentrations presents a significant threat to living organisms. Thus, the development of efficient sensors capable of detecting these metals is of utmost importance. This study explores the utilization of two-dimensional nitrogenated holey graphene (C_2_N) nanosheet as a sensor for toxic transition metals. The C_2_N nanosheet’s periodic shape and standard pore size render it well suited for adsorbing transition metals. The interaction energies between transition metals and C_2_N nanosheets were calculated in both gas and solvent phases and were found to primarily result from physisorption, except for manganese and iron which exhibited chemisorption. To assess the interactions, we employed NCI, SAPT0, and QTAIM analyses, as well as FMO and NBO analysis, to examine the electronic properties of the TM@C_2_N system. Our results indicated that the adsorption of copper and chromium significantly reduced the HOMO–LUMO energy gap of C_2_N and significantly increased its electrical conductivity, confirming the high sensitivity of C_2_N towards copper and chromium. The sensitivity test further confirmed the superior sensitivity and selectivity of C_2_N towards copper. These findings offer valuable insight into the design and development of sensors for the detection of toxic transition metals.

## 1. Introduction

First row transition metals (Sc, Ti, Vn, Cr, Mn, Fe, Co, Ni, Cu and Zn) play undeniable role in various disciplines which include medicine, construction, catalysis, nuclear processes, engineering, and numerous medical applications [1,2,3]. One of the major application accounts for their catalytic properties in isomerization, hydrogenation, oxidation, polymerization and building small molecules, etc. [4,5] In a biological system, transition metals are associated with most of proteins to perform number of enzymatic processes and transportation to their target [6]. However, high concentration causes toxicity and is hazardous to life [7]. All elements of the series have some level of toxicity, but some are highly toxic while others are moderate [8,9]. The first row transition metals have oxidation state dependent toxicity [10]. Manganese has an interesting chemistry of multiple oxidation states. Being essential nutrition, manganese helps in the production of glucose and the feeding of mitochondria during its maintenance [11]. However, Mn in +2 oxidation state predominates in cellular toxicity [12]. Immoderate exposure of Mn^2+^ causes “Manganism”, which is neurodegenerative disorder which leads to neuronal death [13]. Some general effects of manganese toxicity are memory loss, insomnia, headache, and speech disturbances [14]. Similarly, iron is a good biocatalyst but it also has harmful effects [15]. A high concentration of tissue iron causes numerous pathological states such as heart disease, diabetes, abnormality in immune system and liver disorders [16]. Similarly, Fe^2+^ (ferrous ion) is highly poisonous and has corrosive effects on the gastrointestinal mucosa (GI). Nausea, diarrhea, vomiting, and abdominal pain are the outcomes of iron poisoning [17]. Moreover, macromolecules are also destroyed by the free radicals generated by Fe^2+^ in the presence of oxygen, resulting in cell death [18]. Co^+2^ is likely to be more dangerous than Co^+3^. The ultimate effects of cobalt toxicity in a +2 oxidation state are fever, gastrointestinal distress, heart failure, inflammation, and low thyroid level [19]. According to The International Agency for Research on Cancer (IARC), the risk of cancer from cobalt toxicity is about 30% [20]. Copper acts as a component of allosteric the enzyme and is used in the production of a variety of neurotransmitters [21]. Copper in a +2 oxidation state is associated with liver damage, Wilson disease and insomnia [22]. Excessive intake of copper has adverse toxicological complications. Lost cognition is also observed due to a high copper intake in the general population [23]. The notable functions of zinc in humans include proliferation, protein synthesis, neuronal growth, DNA metabolism, free radical sequestration, cellular division and lipid peroxidation [24]. Zn^+2^ is considered toxic when a surplus amount of it is ingested into the body [25]. An elevated risk of prostatic cancer is caused by high doses of zinc [26]. On the contrary, if the toxicity of chromium is extremely interrelated with its oxidation state, it will be highly toxic if the state of oxidation is higher, and vice versa. Cr^+6^ is the sturdiest oxidizing agent and is considered to be 1000 times more dangerous than chromium in a +3 oxidation state [27]. Chromium is cancer-causing metal; its toxicity is not only accessible to humans—plants and animals are also heavily affected by its consumption [28]. Different surfaces are used for the detection, adsorption, and removal of transition metals in different oxidation states; however, the selectivity of a particular metal in the presence of other transition metals is a challenge. Various sensing materials are used for their detection, including kaolinite, magnetite, polyphenol acetaldehyde resins, activated carbon, graphene oxide, picolinamide (Pi-A)-decorated reduced-graphene oxide (RGO), etc. [29,30,31,32,33,34] Here, we are using a C_2_N nanosheet which is already employed as an electrochemical sensor for the detection of toxic and hazardous organic compounds such as HCN, H_2_S, PH_3_, HF, NCl_3_, COCl_2_, NCl_3_, NBr_3_, NH_3_, NI_3_ and NF_3_ [35]. Additionally C_2_N has shown its high efficacy in the field of optics, batteries, gas sensors and photo catalysis [36]. To the best of our knowledge, the nitrogenated holey graphene nanosheet has not been previously adopted as an electrochemical sensor for transition metals. The C_2_N nanosheet has periodic geometry with standard pore size. C_2_N comprises a highly electron-rich cavity consisting of benzene and pyrazine rings alternatively attached. The pyrazine ring of C_2_N provides us with highly electronegative nitrogen, which coheres to and catches transition metals.

Our theoretical study provides valuable insight into the unique properties of the C_2_N nanosheet and its potential applications as a sensor. However, we also acknowledge that experimental studies are required to validate our theoretical findings and to address the practical challenges associated with the use of C_2_N nanosheets, for example, improving the structural stability of C_2_N nanosheets is crucial for their long-term use, as they can be unstable under certain conditions, such as high temperature or when exposed to moisture. Developing methods for the precise control of the size and thickness of C_2_N nanosheets is important for optimizing their properties and performance. Controlling and minimizing defects and impurities in the structure of C_2_N nanosheets is crucial for improving their chemical and electronic properties, as well as their overall performance. While carbon and nitrogen are generally considered non-toxic, the potential toxicity of C_2_N nanosheets is not well understood. Further research is needed to determine any adverse effects of C_2_N on human health and the environment. It is important to handle and use C_2_N nanosheets with caution and follow the appropriate safety guidelines to minimize the potential risks.

## 2. Results and Discussion

### 2.1. Geometric Optimization

**Figure 1:**
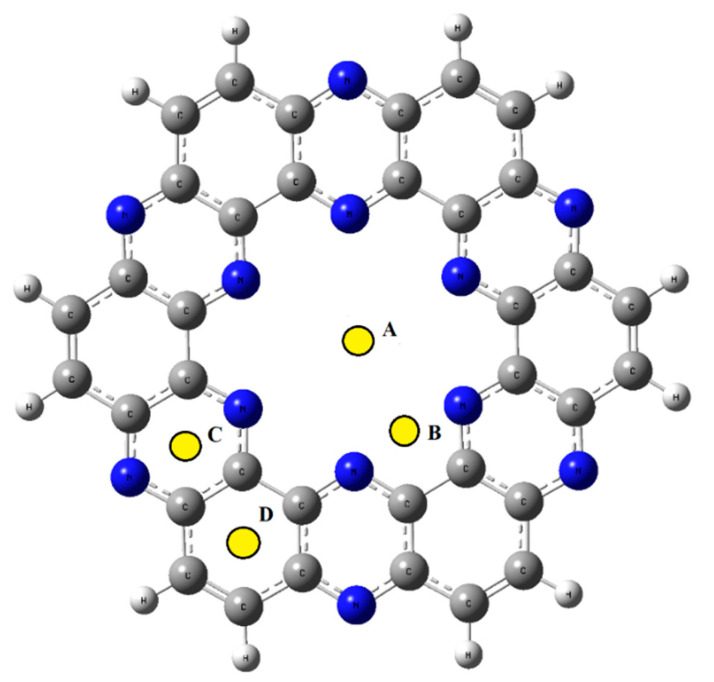
Optimized structure and available adsorption sites of C_2_N surface. A–D shows four possible sites for the adsorption of analytes.

**Figure 2:**
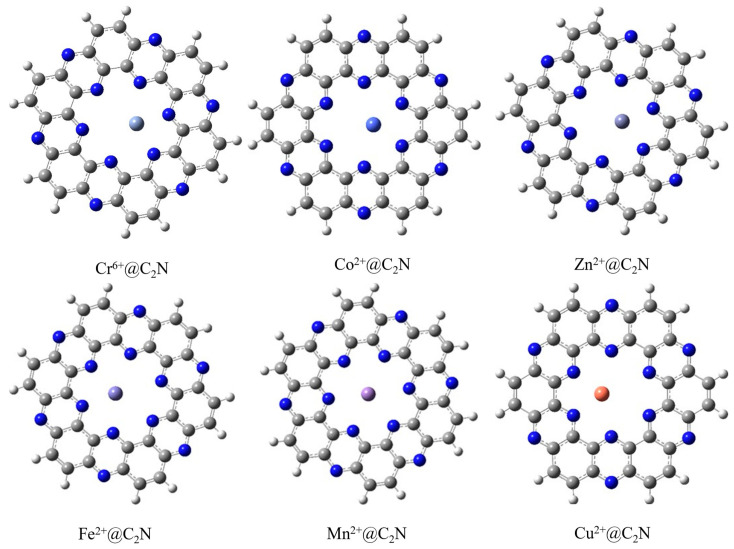
The most stable configuration of TM@C_2_N complexes (Cr^6+^@C_2_N, Co^2+^@C_2_N, Zn^2+^@C_2_N, Fe^2+^@C_2_N, Mn^2+^@C_2_N and Cu^2+^@C_2_N).

The structure of the cluster model of the nitrogenated holey graphene (C2N) nanosheet is shown in Figure 1. A single unit of a C_2_N nanosheet comprises nitrogen atoms which are arranged in a periodical manner to set up a ring diameter of 8.30 Å. The pyrazine ring of C_2_N provided us with highly electronegative nitrogen which acts as an extremely powerful part, cohering and adsorbing toxic transition metals. The single monolayer of C_2_N reflects four possible sites for the adsorption of analytes: (a) in the middle of C_2_N surface (A), (b) in the middle of nitrogen atoms (B), (c) above the pyrazine ring (C), and (d) above the benzene ring (D). To find the most stable geometry with the lowest energy for each TM@C_2_N complex, all possible orientations of each metal ion over carbon nitride surface were explored. All the complexes (Cu^2+^@C_2_N, Mn^2+^@C_2_N, Co^2+^@C_2_N, Fe^2+^@C_2_N, Cr^6+^@C_2_N, Zn^2+^@C_2_N) exhibited the best performance in the central position, i.e., position (A), as it was the most stable and had the lowest energy (Figure 2).

### 2.2. Most Stable Spin State of TM@C_2_N Complexes

In this study, the analytes selected for sensing studies were six metals of a first-row transition series (Cu^2+^, Mn^2+^, Fe^2+^, Zn^2+^, Cr^6+^ and Co^2+^). First, it was essential to determine the most stable spin state of the TM@C_2_N complexes. The optimization of Cu@C_2_N, Mn@C_2_N and Co@C_2_N was carried out in doublet, quartet, sextet, and octet spin states. The relative energies of different spin states for all the complexes are given in Table 1. The most stable spin states obtained for Cu@C_2_N, Mn@C_2_N and Co@C_2_N were doublet, sextet, and quartet spin states, respectively. Similarly, the rest of metal complexes, such as Fe@C_2_N, Cr@C_2_N and Zn@C_2_N, showed quintet, triplet and singlet spin states to the most stable states, respectively.

### 2.3. Interaction Energies

**Figure 3:**
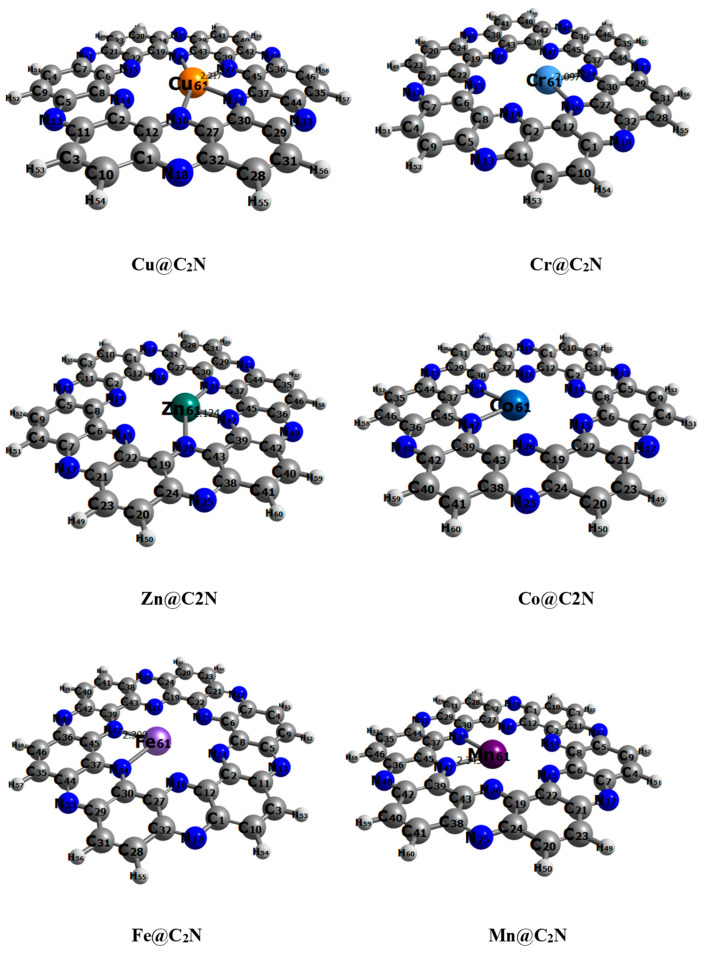
The closest interaction distances D_int_ for TM@C_2_N complexes.

The interaction energies and interaction distances of all the complexes are given in Table 2. In all the complexes, the transition metal interacts with N-atoms of the C_2_N cavity (Figure 3). The value of interaction energy of the Cu^2+^@C_2_N complex is the lowest among all the studied complexes, i.e., −6.6 kcal mol^−1^ along with 2.2 Å of interaction distance between closest interacting (Cu-62….N-47) atoms. The E_int_ and D_int_ (Cr-61…N-34) in the Cr^6+^@C_2_N complex are −9.2 kcal mol^−1^ and 2.09 Å, respectively. In the case of Zn^2+^@C_2_N, Fe^2+^@C_2_N and Co^2+^@C_2_N complexes, the E_int_ is −15.9, −25.9 and −20.7 kcal mol^−1^, respectively. The interaction energy of Mn^2+^@C_2_N (−43.1 kcal mol^−1^) is greater than the rest of the five complexes showing chemisorption; it interacts at site A of the C_2_N cavity with an interaction distance of 2.34 Å.

The interaction energy results of the TM@C_2_N complexes support the existence of a physisorption mechanism, except for Mn@C_2_N and Fe^2+^@C_2_N which show chemisorption. The interaction energy trends observed for TM@C_2_N complexes are Cu@C_2_N > Cr@C_2_N > Zn@C_2_N > Co@C_2_N > Fe@C_2_N > Mn@C_2_N, respectively. The results of the interaction energy indicate that C_2_N can accommodate transition metals on its surface, but the highest interaction energy was seen for the Mn@C_2_N complex (−43.1 kcal mol^−1^).

### 2.4. Natural Bond Orbital Analysis (NBO)

The analysis of natural bond orbital reveals the ability of the sensor to detect the toxic transition metals. The transfer of charge, as well as the direction of charge transfer, was determined using NBO analysis. The calculated charge transfer is listed in Table 3. The transfer of charge may occur from C_2_N to metal or metal to C_2_N. In this study, the charge values on adsorbed metals are 0.871 |𝑒| (Cu), 1.640 |𝑒| (Fe), 1.672 |𝑒| (Mn), 1.652 |𝑒| (Zn), 0.861 |𝑒| (Co) and 1.79 |𝑒| (Cr). The Q_NBO_ values of TM@C_2_N complexes show that charges shifted from analytes (transition metals) to the C_2_N nanosheet, as evidenced by the highly electron-rich cavity of C_2_N (due to the presence of electronegative nitrogen) and the positive charge of the metals. The following order of charge transfer was observed in the complexes: Cr^6+^@C_2_N ˃ Mn^2+^@C_2_N ˃ Zn^2+^@C_2_N ˃ Fe^2+^@C_2_N > Cu^2+^@C_2_N > Co^2+^@C_2_N.

### 2.5. Frontier Molecular Orbital Analysis (FMO)

**Figure 4:**
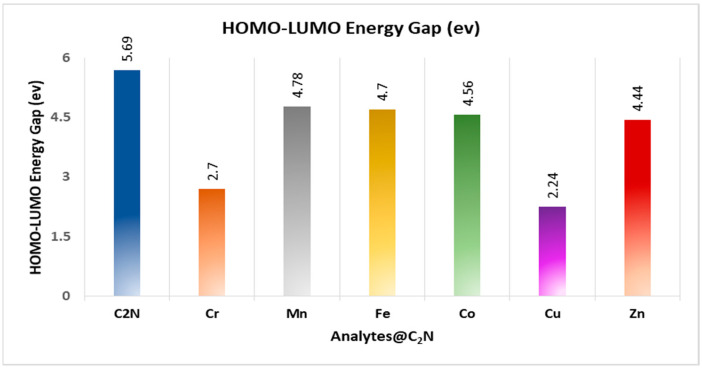
Graphical representation of H–L gap of TM@C_2_N.

**Figure 5:**
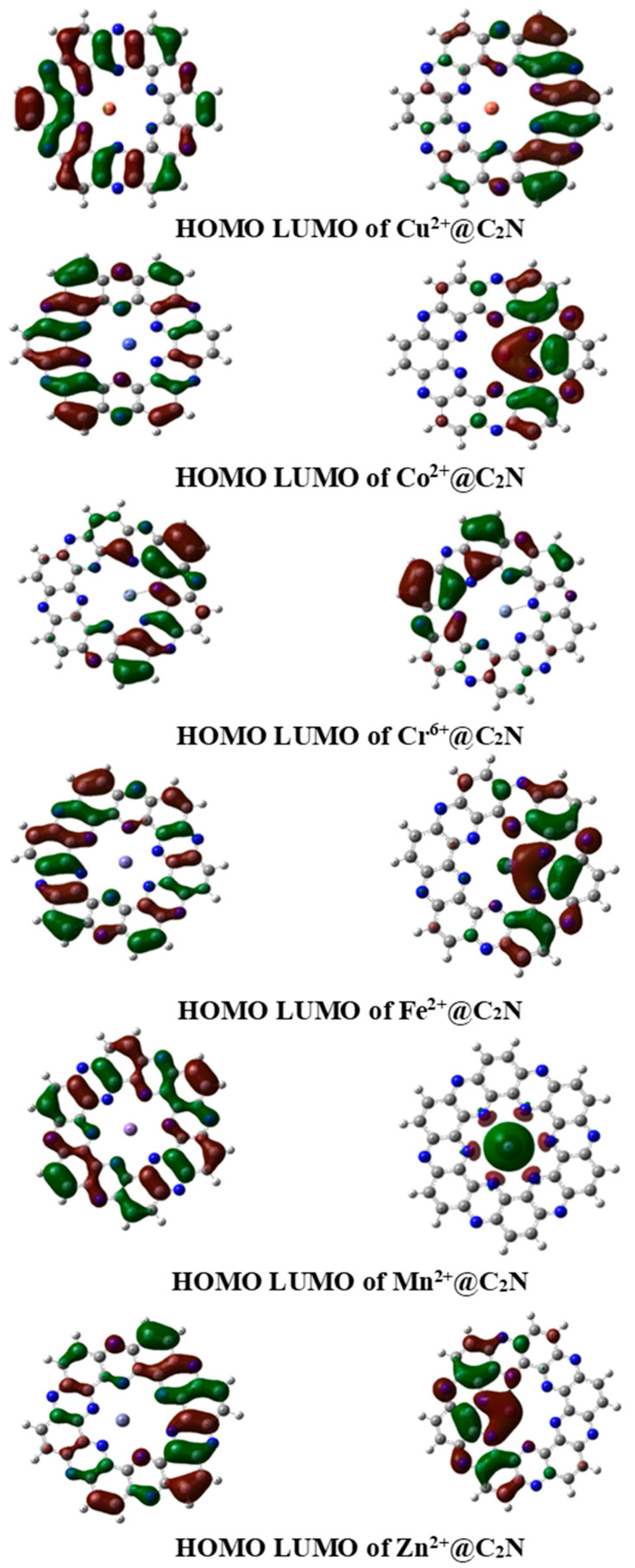
3D isosurfaces of HOMO and LUMO of TM@C_2_N complexes.

The reactivity of interacting substances is significantly defined by frontier molecular orbital analysis (FMO). The energy gap obtained by taking the difference between the highest occupied molecular orbital (HOMO) and the lowest unoccupied molecular orbitals (LUMO) greatly influences the conductivity as well as the stability of complexes. The E_HOMO_ and E_LUMO_ of C_2_N are −7.87 eV and −2.17 eV, respectively. The H–L energy gap in C_2_N is 5.71 eV. In TM@C_2_N complexes, the H–L energy gap reduced to 2.24 eV (Cu), 4.71 eV (Fe), 4.80 eV (Mn), 4.44 eV (Zn), 2.70 (Cr) and 4.56 eV (Co) as graphically represented in Figure 4. In the Cu@C_2_N complex, HOMO and LUMO energies were −13.3 eV and −11.06 eV, respectively. A moderate reduction in H–L energy gap was observed for the complexes of Fe, Mn, and Co, i.e., 4.71, 4.80 and 4.56 eV, respectively. However, a remarkable decrease was observed in the E_H–L_ energy gap of Cu^2+^@C_2_N (2.24 eV), which indicates the increased conductivity and sensitivity of C_2_N towards copper. Similarly, the LUMO (−21.35 eV) and HOMO (−24.05) of Cr^6+^@C_2_N were more highly stabilized compared to the bare C_2_N unit (−2.17 eV, −7.870 eV), which caused a notable decrease in the H–L energy gap (2.70 eV). The notable decrease in the E_H–L_ gap evidences the greater sensitivity of C_2_N towards copper and chromium. The appreciable downfall in the HOMO–LUMO energy gap of any substances represents its appreciable sensitivity and selectivity towards toxic transition metals. Moreover, the orbital densities are also analyzed to visualize the interaction behavior of transition metals and the C_2_N surface (Figure 5). All the complexes show totally different orbital densities for LUMO. Except for manganese, the densities of LUMO for the rest of complexes were distributed at different half portions of the C_2_N cavity. In case of Mn@C_2_N, the LUMO was entirely located on manganese metal at the center of cavity, whereas the density of HOMO was present as seen in the C_2_N unit. The highest decline in the H–L energy gap was observed in the Cu@C_2_N having the maximum conductivity. Therefore, Cu tends to enhance the conductivity of the carbon nitride surface as compared to the other selected metals.

### 2.6. Non-Covalent Interactions (NCI)

**Figure 6:**
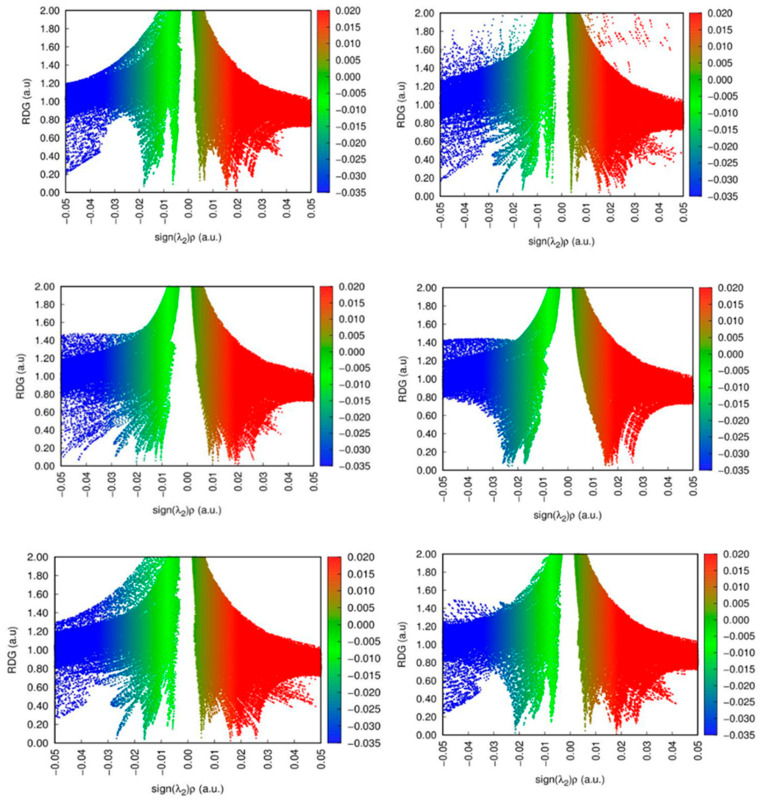
2D RDG graphs of TM@C_2_N complexes (Cr^6+^@C_2_N, Co^2+^@C_2_N, Zn^2+^@C_2_N, Fe^2+^@C_2_N, Mn^2+^@C_2_N and Cu^2+^@C_2_N).

NCI analysis reveals the nature of interactions between the analyte and the surface through RDG graphs and 3D isosurfaces. The RDG graph is based on the following equation:

$$\mathrm{RDG}\left(s\right)=\frac{1}{2{\left(3\pi^{2}\right)}^{\frac{1}{3}}}\;\frac{\nabla \rho }{\rho^{\frac{4}{3}}}$$

where ∇ρ and ρ are electronic density gradient and electronic density, respectively. The color scheme of NCI graphs comprises different types of interactions; the steric repulsion is reported in the red color, while weak and strong interactions are represented by the green and blue colors, respectively. In the 3D isosurfaces of all the TM@C_2_N complexes, the patches of interactions are mainly shown in the middle part, i.e., the center of the ring; the dotted patches reflect weak interactions, whereas the thicker patches show the strong interactions. In all TM@C_2_N complexes, the appearance of green surfaces of different intensities between the metals and C_2_N surface indicates the existence of strong and weak van der Waals interactions. In all the complexes, steric clashes are observed from the presence of thicker patches of red color; these repulsive forces are observed due to the presence of the delocalized electrons present in the metals as well as the nitrogen atom of the pyrazine rings. In RDG plots as shown in the Figure 6, the TM@C_2_N presents a variety of greenish peaks between −0.02 and 0.01 a.u. A dispersive light-bluish and the greenish spike (nearly −0.02 a.u.) implies the presence of strong non-bonding interactions.

### 2.7. QTAIM Analysis

**Figure 7:**
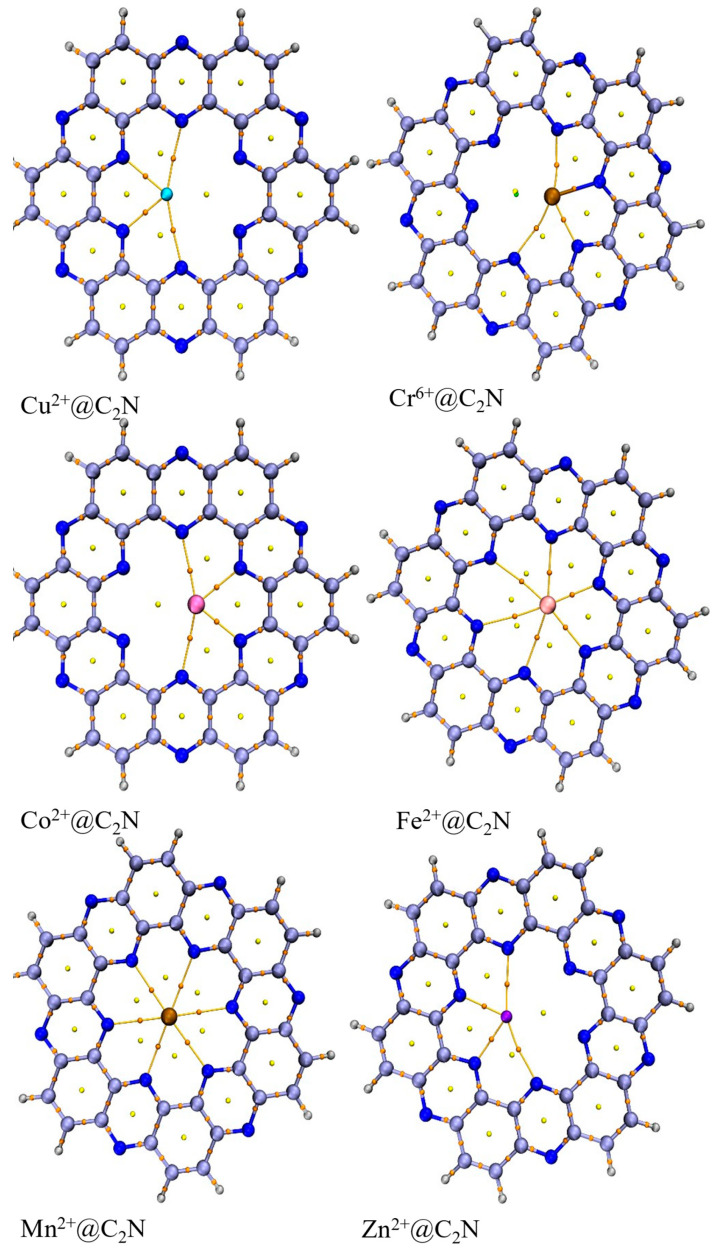
The BCPs obtained using a QTAIM analysis of TM@C_2_N complexes.

In QTAIM analysis, the bond nature between analyte and complex depends on bond-critical point (BCP). BCP’s are further classified into five components, i.e., electronic density (ρ), potential energy density *V*(*r*), energy density *H*(*r*), Laplacian of electron density (∇^2^ ρ) and kinetic energy density *G*(*r*). The bond-critical point (BCP) can be more distantly elucidated by the following equation:

$$H\left(r\right)=G\left(r\right)+V\left(r\right)$$

which shows that the sum of the potential energy and kinetic energy density is equal to the electron density. The value of *H*(*r*) > 0 and *H*(*r*) < 0 indicates the presence of closed-shell and shared-shell interactions, respectively. The bond-critical point (BCP) results of electronic density (ρ) and Laplacian (∇^2^ ρ) are given in Table 4 and the BCPs are depicted in Figure 7. The geometry of Cu@C_2_N complexes consists of four BCPs. The bond-critical point values of ρ ranges from 0.02 to 0.05 a.u. and ∇^2^ρ from 0.07 to 0.19 a.u. Among four BCPs values, two interactions (N23 --- Cu30 and N36 --- Cu30 of C_2_N and Cu) contain the highest value of electronic density (ρ), 0.05 a.u. In Cu@C_2_N, the values of electronic density (ρ) are less than 0.1, which indicate the presence of weak van der Waals interactions, as confirmed by a 3D isosurface of an NCI plot. The highest number of BCPs obtained among the studied systems was six in the cases of Fe^2+^@C_2_N and Mn^2+^@C_2_N. The values of 0.02 a.u to 0.35 a.u for ρ, and −0.10 a.u to −0.97 a.u for ∇^2^ρ, were obtained for Fe^2+^@C_2_N. In case of Fe^2+^@C_2_N, one of the values of electronic density (ρ) was less than zero, which indicates the existence of electrostatic interactions, as confirmed by SAPT0 analysis. Similarly, the electronic density (ρ) of Zn^2+^@C_2_N (0.2 to 0.4 a.u) is greater than 0.1, which confirms the electrostatic interactions between zinc and the C_2_N nanosheet. For Cr^6+^@C_2_N and Co^2+^@C_2_N, the values of electronic density confirm the presence of van der Waals interactions, i.e., ρ < 0.1 as confirmed by the greenish patches in the RDG graph. The outcomes of QTAIM analysis were in great accordance with the NCI and SAPT0 analyses.

### 2.8. SAPT0 Analysis

Symmetry-adapted perturbation theory (SAPT) provides a quantitative analysis of the noncovalent interaction between two entities through the perturbative approach by directly computing the interaction energy as a perturbation to the Hamiltonian of the individual monomers instead of the supermolecular approach. The division of interaction energy into different components such as dispersion, exchange, electrostatic and induction has been performed using SAPT (E_int_ SAPT = E_ele_ + E_ind_ + E_disp_ + E_exch_). The interpretation of SAPT0 analysis is helpful for providing an explanation of the nature of interactions between the analytes (metals) and the C_2_N unit, and for quantifying the chemical bonds. The interactions of SAPT0 for six complexes are shown in Table 5. The components of SAPT0, which are negative, reveal the presence of attractive interactions between the C_2_N units and the transition metals. The exchange part contains the positive energies, which indicates the presence of repulsive interactions between the C_2_N unit and the analytes. As shown in Table 5, the negative energies of SAPT0 among all the complexes (TM@C_2_N) denote the presence of attractive interactions. The interaction energies obtained in SAPT0 studies of metal complexes were −255.09 kcal/mol (Cu^2+^@C_2_N), −131.88 kcal/mol (Fe^2+^@C_2_N), −283.35 kcal/mol (Mn^2+^@C_2_N), −306.20 kcal/mol (Zn^2+^@C_2_N), −174 kcal/mol (Co^2+^@C_2_N) and −5145.7 kcal/mol (Cr^6+^@C_2_N). The highest contribution towards the total SAPT0 was observed for E_elec._ Hence, electrostatic interactions dominate and stabilize the complexes. The findings of the SAPT0 analysis are consistent with the NCI and QTAIM analyses. The trend in supermoleculor interaction energy (without solvent) is exactly followed by the E_SAPT0_. The trend for E_SAPT0_ is Cr@C_2_N > Zn@C_2_N > Mn@C_2_N > Cu@C_2_N > Co@C_2_N < Fe@C_2_N.

### 2.9. Electrical Conductivity and Sensitivity Analysis

Electrical conductivity (σ) is calculated for pristine C_2_N and TM@C_2_N complexes at 300 K and the σ values are given in Table 6. The conductivity of TM@C_2_N complexes show a marked increase as compared to pristine C_2_N. In particular, Cu^2+^@C_2_N and Cr^6+^@C_2_N conductivities increase largely when compared to other TM@C_2_N complexes. This large increase in conductivity can be converted to an electrical signal. Therefore, it can be concluded that the nitrogenated holey graphene C2N may be a promising electronic sensor for the detection of Cu and Cr. To further confirm the higher sensitivity of C_2_N towards toxic transition metals, the sensing characteristics of C_2_N were quantitatively analyzed using a sensitivity (S) test. Sensitivity is the response of a sensor towards analyte exposure. A high value of S means the material is an excellent sensor for the particular analyte. The sensitivity of the C_2_N nanosheet is found to be 142.63, 10.79, 0.09, 0.69, 107.30, and 51.90 towards Cu, Fe, Mn, Zn, Cr, and Co, respectively. Cu adsorbed on the C_2_N complex shows the highest S value, which indicates that among the mixture of analytes, C_2_N is the most sensitive and selective towards Cu.

## 3. Computational Methodology

The level of theory employed for geometry optimization is M05-2X/6-31+G (d, p) [37,38]. In our investigation of C_2_N–transition metal complexes, non-covalent interactions are involved, and therefore, we have employed the hybrid meta-exchange-correlation functional M05-2X. The choice of M05-2X functional is based on its accuracy in describing non-covalent interactions, as demonstrated by benchmark studies such as the one by Burns et al. [39] and other studies reported in the literature on the non-covalent interactions [40,41,42,43]. Additionally, the choice of an appropriate basis set is a crucial factor in computational simulations. To this end, we have utilized the double zeta basis set 6-31+G(d,p), which includes diffuse and polarized functions and strikes a balance between accuracy and computational efficiency [44]. The interaction energies between C_2_N and transition metals were calculated by using the following equation:

$$E_{int}=E_{TM@C_{2}N}-\left(E_{C_{2}N}+E_{TM}\right)$$

where $E_{TM@C_{2}N}$, $E_{C_{2}N}$ and $E_{TM}$ are the interaction energies of C_2_N–transition metal complexes, pristine C_2_N surface, and isolated transition metal, respectively. To find the lowest energy structure, each metal is placed in different possible orientations and the results are visualized using Gaussview 5.0 [45]. To validate the true minima’s nature of the TM@C_2_N complexes, vibrational frequencies were examined for the presence of imaginary frequencies. An NCI analysis was carried out using VMD and Multiwfn software for an improved evaluation of the interactive sites between C_2_N and the selected transition metals [46,47]. The electronic properties, such as the natural bond orbital (NBO) and frontier molecular orbital (FMO) analyses, were performed at the same level of theory used for optimization. The non-covalent interactions between C_2_N and transition metals were quantified by bond-critical point using QTAIM analysis [48]. The interaction energies between transition metals and the C_2_N nanosheet were also analyzed using SAPT0 (symmetry-adaptation perturbation theory). SAPT0 analysis illustrates four types of interactions: electrostatic (ΔE_elst_), exchange (ΔE_exh_), induction (ΔE_ind_) and dispersion (ΔE_dis_) [49]. The equation for ΔE_int_ through SAPT0 is as follows:

ΔE_int_ = ΔE_elst_ + ΔE_exh_ + ΔE_ind_ + ΔE_dis_

All the SAPT calculations were performed using PSI4 1.6 software [50].

## 4. Conclusions

This study aimed to systematically investigate the ability of a two-dimensional nitrogenated holey graphene (C_2_N) nanosheet to detect toxic transition metals (Cu^2+^, Mn^2+^, Fe^2+^, Zn^2+^, Cr^6+^, and Co^2+^) using DFT calculations. The interaction energies between C_2_N and each of the transition metals were calculated, with values of −6.6, −43.1, −20.7, −25.9, −9.2, and −15.9 kcal mol^−1^ for Cu^2+^@C_2_N, Mn^2+^@C_2_N, Co^2+^@C_2_N, Fe^2+^@C_2_N, Cr^6+^@C_2_N, and Zn^2+^@C_2_N, respectively. These results suggest that the adsorption of the transition metals on C_2_N is mainly due to physisorption, except for Mn and Fe, which are chemisorbed. The dominant interaction between C_2_N and the metals was found to be the electrostatic force of attraction, which stabilizes the TM@C_2_N complexes. The computed E_H–L_ gap of the C_2_N nanosheet was found to decrease significantly upon the adsorption of Cu^2+^ and Cr^6+^. The NCI plots and QTAIM analysis showed the presence of strong and weak van der Waals interactions between C_2_N and the metals. The increase in the electrical conductivity of Cu^2+^@C_2_N and Cr^6+^@C_2_N, as compared to the pristine C_2_N, indicates the superior sensitivity of C_2_N towards these metals. The sensitivity (S) test also confirms the higher sensitivity and selectivity of C_2_N towards Cu^2+^. In conclusion, the results of this study suggest that the two-dimensional nitrogenated holey graphene (C_2_N) nanosheet could be an effective sensor for detecting copper (II) and hexavalent chromium. The findings may also provide valuable insights into the design and development of sensors for detecting other toxic metals.

## Figures and Tables

**Table 1:** Relative energies in kcal mol^−1^ for the spin states of TM@C_2_N complexes.

Spin States	Complexes		
	Cu^2+^@C_2_N	Co^2+^@C_2_N	Mn^2+^@C_2_N
Doublet	0.0	23	68
Quartet	37	0.0	69
Sextet	85	41	0.0
Octet	136	89	46
	Cr^6+^@C_2_N	Fe^2+^@C_2_N	Zn^2+^@C_2_N
Singlet	80	60	0.0
Triplet	0.0	21	28
Quintet	0.4	0.0	29
Septet	6.8	35	30

**Table 2:** Interaction energies (kcal mol^−1^) of the most stable TM@C_2_N complexes.

TM@C_2_N (Solvent = Water)	Interaction Energy (kcal mol^−1^)	Interacting Atoms	Interaction Distance (Å)
Cu^2+^@C_2_N (Doublet)	−6.6	Cu-61………N-47	2.21
Cr^6+^@C_2_N (Quintet)	−9.2	Cr-61………..N-34	2.09
Zn^2+^@C_2_N (Singlet)	−15.9	Zn-61………N-47	2.12
Co^2+^@C_2_N (Quartet)	−20.7	Co-61………N-34	2.20
Fe^2+^@C_2_N (Sextet)	−25.9	Fe-61………N-47	2.30
Mn^2+^@C_2_N (Triplet)	−43.1	Mn-61……..N-47	2.34

**Table 3:** The E_HOMO_, E_LUMO_, E_H–L_ gap (in eV) of TM@C_2_N complexes and charges on metals |𝑒|.

Complexes	E_HOMO_	E_LUMO_	E_H–L_ (eV)	Q_NBO_
C_2_N	−7.870	−2.17	5.71	-
Cu^2+^@C_2_N	−13.30	−11.06	2.24	0.871
Fe^2+^@C_2_N	−13.22	−8.51	4.71	1.640
Mn^2+^@C_2_N	−13.27	−8.48	4.80	1.672
Zn^2+^@C_2_N	−13.17	−8.73	4.44	1.652
Cr^6+^@C_2_N	−24.05	−21.35	2.70	1.79
Co^2+^@C_2_N	−13.20	−8.637	4.56	0.861

**Table 4:** The values of topological parameter of BCPs of TM@C_2_N complex obtained from QTAIMs analysis.

Analytes@C_2_N	C_2_N—Analyte	BCP	ρ	∇ ^2^ρ	G (r)	V (r)	H (r)	V(r)/G(r)
Cu^2+^@C_2_N	N18-	96	0.02	0.07	0.01	−0.01	0.001	1.0
	N23-	99	0.05	0.19	0.06	−0.08	−0.014	1.3
	N36-	112	0.05	0.19	0.06	−0.07	−0.014	1.1
	N44-	117	0.02	0.07	0.01	−0.04	0.001	4
Fe^2+^@C_2_N	N18-	97	0.35	−0.10	0.34	−0.94	−0.59	2.7
	N23-	100	0.33	−0.97	0.11	−0.48	−0.36	4.3
	N25-	104	0.29	−0.78	0.07	−0.34	−0.27	4.8
	N37-	115	0.30	−0.82	0.08	−0.37	−0.29	4.6
	N39-	119	0.34	−0.10	0.31	−0.89	−0.57	2.8
	N-44	122	0.02	0.18	0.04	−0.03	0.008	0.7
Zn^2+^@C_2_N	N20-	97	0.40	−0.010	0.34	−0.90	−0.6	2.6
	N21-	101	0.29	−0.779	0.078	−0.35	−0.3	4.4
	N33-	109	0.29	−0.114	0.033	0.35	−0.3	10
	N42-	115	0.302	−0.82	0.086	−0.37	−0.2	4.3
Mn^2+^@C_2_N	N19-	97	0.35	−0.10	0.34	−0.94	−0.6	2.7
	N22-	101	0.293	−0.779	0.078	−0.35	−0.3	4.4
	N28-	106	0.292	−0.11	0.032	−0.35	−0.3	11
	N34-	111	0.28	−0.76	0.073	−0.33	−0.3	4.5
	N40-	116	0.33	−0.99	0.122	−0.49	−0.4	4.0
	N43-	120	0.015	0.045	0.010	−0.009	0.008	0.9
Cr^6+^@C_2_N	N18-	94	0.016	0.033	0.010	−0.012	−0.002	1.2
	N23-	97	0.053	0.29	0.071	−0.069	0.003	0.9
	N36-	108	0.069	0.375	0.095	−0.097	−0.002	1.0
	N44-	112	0.026	0.068	0.020	−0.024	0.004	1.2
Co^2+^@C_2_N	N18-	98	0.02	0.05	0.016	−0.019	−0.003	1.1
	N26-	103	0.057	0.18	0.059	−0.072	−0.013	1.2
	N39-	116	0.057	0.18	0.059	−0.072	−0.013	1.2
	N44-	117	0.021	0.0561	0.016	−0.019	−0.002	1.1

**Table 5:** The SAPT0 analysis (in kcal mol^−1^) of TM@C_2_N complexes.

TM@C_2_N	E_elec_	E_exch_	E_Ind_	E_disp_	E_SAPT0_
Cu^2+^@C_2_N	−167.46	41.33	−121.82	−7.15	−255.09
Fe^2+^@C_2_N	−163.28	38.03	0.3244	−6.96	−131.88
Mn^2+^@C_2_N	−156.84	24.55	−144.44	−6.62	−283.35
Zn^2+^@C_2_N	−173.33	45.06	−170.61	−7.31	−306.20
Co^2+^@C_2_N	−172.68	54.46	−48.38	−7.82	−174.43
Cr^6+^@C_2_N	−463.26	81.73	−4759.46	−4.76	−5145.76

**Table 6:** Work function (Φ), sensitivity S (%), and conductivity (σ) values of TM@C_2_N complexes.

Complexes	Φ	S (%)	σ
C_2_N	5.02	-	1.09 × 10^−48^
Cu^2+^@C_2_N	12.18	142.63	1.53 × 10^−19^
Fe^2+^@C_2_N	10.86	10.79	2.74 × 10^−40^
Mn^2+^@C_2_N	10.87	0.09	4.80 × 10^−41^
Zn^2+^@C_2_N	10.95	0.69	5.08 × 10^−38^
Cr^6+^@C_2_N	22.7	107.30	2.09 × 10^−23^
Co^2+^@C_2_N	10.92	51.90	4.98 × 10^−39^

## Data Availability

All data are provided in the manuscript.
